# Electrophysiological characterization of a Ca_v_3.2 calcium channel missense variant associated with epilepsy and hearing loss

**DOI:** 10.1186/s13041-023-01058-2

**Published:** 2023-09-21

**Authors:** Robin N. Stringer, Leos Cmarko, Gerald W. Zamponi, Michel De Waard, Norbert Weiss

**Affiliations:** 1 Department of Pathophysiology, Third Faculty of Medicine, Charles University, Prague, Czech Republic; 2 Institute of Organic Chemistry and Biochemistry, Czech Academy of Sciences, Prague, Czech Republic; 3 Institute of Biology and Medical Genetics, First Faculty of Medicine, Charles University, Prague, Czech Republic; 4 Nantes Université, CNRS, INSERM, l’Institut du Thorax, Nantes, France; 5 Department of Clinical Neurosciences, Alberta Children’s Hospital Research Institute, Hotchkiss Brain Institute, Cumming School of Medicine, University of Calgary, Calgary, Canada

**Keywords:** Ion channels, Calcium channels, T-type channels, *CACNA1H*, Ca_v_3.2, Mutation, Epilepsy, Hearing, Channelopathy

## Abstract

T-type calcium channelopathies encompass a group of human disorders either caused or exacerbated by mutations in the genes encoding different T-type calcium channels. Recently, a new heterozygous missense mutation in the *CACNA1H* gene that encodes the Ca_v_3.2 T-type calcium channel was reported in a patient presenting with epilepsy and hearing loss—apparently the first *CACNA1H* mutation to be associated with a sensorineural hearing condition. This mutation leads to the substitution of an arginine at position 132 with a histidine (R132H) in the proximal extracellular end of the second transmembrane helix of Ca_v_3.2. In this study, we report the electrophysiological characterization of this new variant using whole-cell patch clamp recordings in tsA-201 cells. Our data reveal minor gating alterations of the channel evidenced by a mild increase of the T-type current density and slower recovery from inactivation, as well as an enhanced sensitivity of the channel to external pH change. To what extend these biophysical changes and pH sensitivity alterations induced by the R132H mutation contribute to the observed pathogenicity remains an open question that will necessitate the analysis of additional *CACNA1H* variants associated with the same pathologies.

**Supplementary Information:**

The online version contains supplementary material available at 10.1186/s13041-023-01058-2.

Mutations in the *CACNA1H* gene that encodes the Ca_v_3.2 T-type calcium channel are risk factors for a number of human channelopathies including epilepsy [1], primary aldosteronism [2], autism spectrum disorder [3, 4], amyotrophic lateral sclerosis [5, 6], congenital amyotrophy [7], and trigeminal neuralgia [8, 9]. Recently, Algahtani and colleagues reported a new heterozygous missense mutation in a 50-year-old female patient with a clinical condition involving epilepsy and hearing loss which appears to be the first *CACNA1H* variant to be associated with sensorineural hearing alterations [10]. This mutation results in the substitution of an arginine at position 132 with a histidine (R132H) in the proximal extracellular end of the second transmembrane helix of Ca_v_3.2 (Fig. 1a) and has not yet been reported in the gnomAD database (https://gnomad.broadinstitute.org/). Molecular simulation using the AlphaFold-generated model of the human Ca_v_3.2 channel shows that replacement of the arginine 132 with a histidine leads to an additional hydrogen bond with methionine 119 of the first transmembrane helix (Fig. 1a) that has the potential to alter the gating of the channel. In addition, a histidine residue has a highly variable pKa value depending of its direct environment indicating that its charge may vary subtly as a function of external pH. To challenge this hypothesis, we assessed the functional impact of the R132H variant on the biophysical properties of Ca_v_3.2 using patch-clamp recordings in tsA-201 cells bathed in 5 mM barium as the charge carrier (see Additional file 1). Both cells expressing Ca_v_3.2 wild-type (WT) and R132H mutant channels displayed characteristic low-voltage activated T-type currents (Fig. 1a, b). A significant 40% (*p* = 0.0285) increase of the maximal macroscopic T-type conductance (*G*_max_) was observed in cells expressing the R132H channel variant (0.52 ± 0.06 nS/pF, n = 26) compared to cells expressing the WT channel (0.37 ± 0.03 nS/pF, n = 24) (Fig. 1d) without any alteration of the voltage dependence of activation (Fig. 1e) or steady-state inactivation (Fig. 1f). An additional significant (*p* = 0.0342) slowing of the time constant (τ) of recovery from inactivation was observed for R132H channels (467 ± 21 ms, n = 18) compared to WT channels (284 ± 34 ms, n = 10) (Fig. 1g) while fast activation and inactivation kinetics of the current remained unaltered (Fig. 1h).

**Fig. 1:**
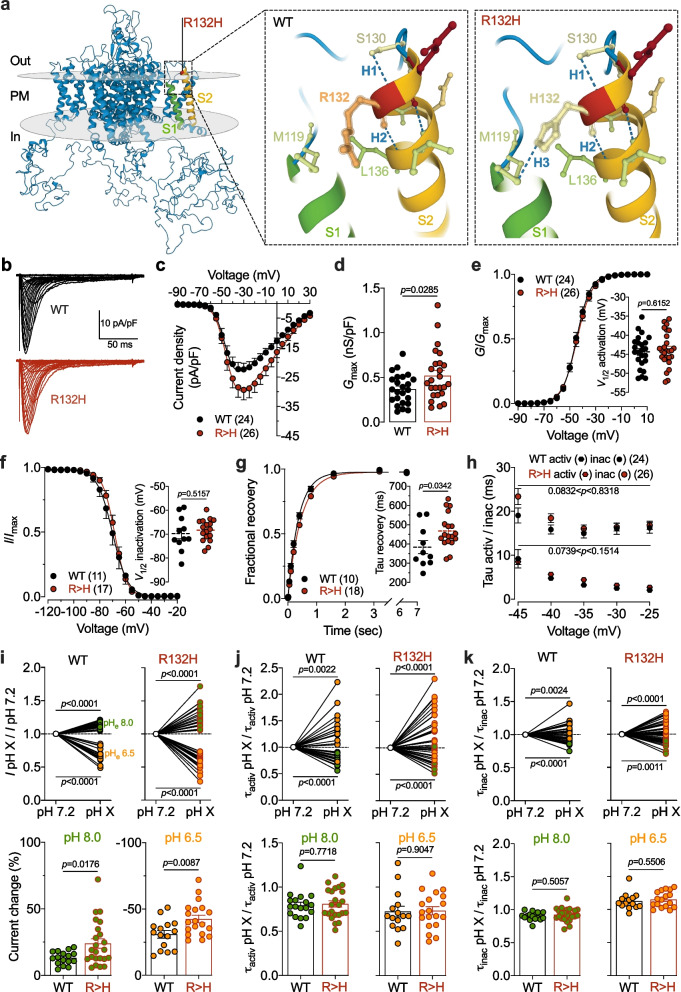
Functional properties of the Ca_v_3.2 R132H variant associated with epilepsy and hearing loss. **a** AlphaFold model of the human Ca_v_3.2 channel showing the location of the R132H mutation (left panel). In this model, the arginine (R) 132 is located within the extracellular-exposed proximal end of the second transmembrane helix (S2) of Ca_v_3.2 and forms two intra-helix hydrogen bonds with serine (S) 130 and leucine (L) 136 (left panel). Substitution of the R132 with a histidine (H) residue creates an additional hydrogen bond with methionine (M) 119 located within the first transmembrane helix (S1) of the channel. **b** Representative sets of whole-cell T-type current traces recorded in tsA-201 cells expressing Ca_v_3.2 wild-type (WT) (black traces) and R132H variant (red traces). Currents were elicited by depolarizing steps to values ranging between − 90 mV and + 30 mV from a holding potential of − 100 mV. **c** Corresponding mean current/voltage (*I*/*V*) relationships. The continuous lines represent the fit of the *I*/*V* curves with the modified Boltzmann Eq. (1). **d** Corresponding mean maximal macroscopic conductance values (*G*_max_) obtained from the fit of the *I*/*V* curves. **e** Corresponding mean normalized voltage dependence of activation fitted (continuous lines) with the modified Boltzmann Eq. (2). The inset shows the mean half-activation potential values obtained from the fit of the conductance curves. **f** Mean normalized voltage dependence of steady-state inactivation fitted with the two-state Boltzmann Eq. (3). The inset represents the mean half-inactivation potential values obtained from the fit of the inactivation curves. **g** Mean normalized recovery from inactivation kinetic fitted with the single-exponential function (4). Inset shows the mean time constant values obtained from the fit of the recovery from inactivation curves. **h** Mean time constant of fast activation (diamond symbols) and inactivation (round symbols) kinetics of T-type currents as a function of the membrane potential. **i** Relative change in peak current amplitude in response to extracellular pH alkalinization (pH_e_ 8.0, green symbols) and acidification (pH_e_ 6.5, orange symbols) from physiological pH_e_ 7.2 (top panels) as well as corresponding mean current change amplitude values (bottom panels). T-type currents were elicited by repetitive depolarizing steps to -20 mV from a holding potential of -100 mV. **j, k** Legend same as (**i**) but for T-type current activation and inactivation kinetics. Data are presented as mean ± S.E.M. and statistical analysis was performed using a two-tailed Student’s *t* test

Next, we aimed to assess the effect of extracellular pH (pH_e_) on the regulation of the channels. Indeed, histidine residues theoretically bear a partial charge at physiological pH, although this is largely influenced by the direct environment of the residue, and therefore act as [H^+^] sensor as a result of protonation. Protonation may in turn mediate modulatory effects on voltage-gated channels, including Ca_v_3.2 [11]. Given that the R132H variant implicates the introduction of histidine within the extracellular end of the second transmembrane helix of Ca_v_3.2, we assessed the effects of extracellular pH changes, alkalization (pH_e_ 8.0) and acidification (pH_e_ 6.5), on T-type currents. Consistent with previous results on T-type channels [11, 12], extracellular alkalization and acidification produced a significant increase and decrease of the T-type current, respectively, in both Ca_v_3.2 WT- and R132H-expressing cells (Fig. 1i, top panels). However, these effects were emphasized on Ca_v_3.2 R132H-mediated currents. For instance, alkalization-mediated increase of the T-type current was 82% higher (*p* = 0.0176) in cells expressing the R132H channel (24.0 ± 3.5% increase, n = 23) compared to cells expressing the WT channel (13.1 ± 1.2% increase, n = 16), whereas acidification-mediated decrease of the current was enhanced by 37% greater (*p* = 0.0087) (from − 30.9 ± 2.9% decrease in WT, n = 15, to − 42.5 ± 2.9% for R132H, n = 19) (Fig. 1i, bottom panels). In addition, extracellular alkalization produced an acceleration of the kinetics of current activation and inactivation, whereas acidification produced the exact opposite (Fig. 1j, k, top panels). However, these effects were proportionally similar between WT and R132H channels (Fig. 1j, k, bottom panels).

Previous studies in animal models have documented the importance of T-type channels in the functioning of the auditory system. For instance, Ca_v_3.2 channels are highly expressed in mouse spiral ganglion neurons (SGN) where they are necessary for spatiotemporal auditory processing [13]. However, they also exhibit age-dependent increases in expression levels that are causally associated with SGN degeneration, whereas T-type channel blockers are protective against age-related SGN and hearing loss [14]. Here, we showed that the Ca_v_3.2 R132H mutation causes mixed alterations of the channel as evident from an increase in current density (that can be attributed to an alteration of the single channel gating properties and/or an increased expression of Ca_v_3.2 at the cell surface) consistent with a gain-of-channel function. There is also a slowing of the recovery from inactivation which is consistent with a loss-of-function of the channel. However, the extent to which this loss-of-gating will manifest under physiological conditions will largely depend on the firing properties of nerve cells expressing the mutant channel. In addition, we illustrate that the R132H mutation enhances the impact of pH_e_ regulation of the channel. While the alterations may seem relatively mild, they have the merit to be observed and will require further experimentation to define their meaning in terms of pathogenicity. Clearly, there is evidence that alteration of pH homeostasis in response to primary metabolic disorders such as renal tubular acidosis is often accompanied with sensorineural hearing alterations [15]. In such context, altered pH_e_-dependent modulation of Ca_v_3.2 by the R132H mutation may represent a risk factor for hearing loss. Likewise, there is evidence that brain pH levels are significantly increased in experimental animal models of epilepsy [16–19] and patients [20] and precipitates the development of seizures. Therefore, it is a possibility that alkalinization-meditated increase of Ca_v_3.2 R132H currents may also exacerbate seizures. An interesting consideration is whether a primary epilepsy could be the initiator of subsequent hearing loss in the patient carrying the Ca_v_3.2 R132H mutation.

In conclusion, it is premature to recommend classifying the Ca_v_3.2 R132H mutation as disease-causing variant at this stage in the absence of a larger number of variants causing the same pathologies. Moreover, since our functional analysis was performed in a recombinant expression system, there remains the possibility that the R132H mutation may exhibit a more pronounced phenotype in a native neuronal environment, and additional analysis will help to fully comprehend to which extent this mutation alters Ca_v_3.2 channel function in the context of auditory function and epilepsy.

### Supplementary Information

**Additional file 1.** Supplementary material and methods.

## Data Availability

All data generated or analyzed during this study are included in this published article and its additional files.

## Abbreviations

Ca_v_: Voltage-gated calcium channel
*G*_max_: Maximal macroscopic conductance
