# Supplementary material for: Electrophysiological characterization of a Cav3.2 calcium channel missense variant associated with epilepsy and hearing loss
Source: Mol Brain. 2023 Sep 21;16:68. doi: 10.1186/s13041-023-01058-2 (PMC10515227; doi:10.1186/s13041-023-01058-2)
Supplement: Supplementary file 1 — Additional file 1. Supplementary material and methods. [file 13041_2023_1058_MOESM1_ESM.docx]

**Supplementary information**

**Supplementary Materials and Methods**

***Plasmid cDNA constructs and site-directed mutagenesis***

The Ca_v_3.2 R132H variant was generated by site-directed mutagenesis performed by GenScript using the wild-type human Ca_v_3.2 (containing exon 26) in pcDNA3.1. The fidelity of the construct was confirmed by full-length sequencing of the coding region.

***Cell culture and heterologous expression***

Human embryonic kidney tsA-201 cells were grown in DMEM medium supplemented with 10% fetal bovine serum and 1% penicillin/streptomycin (all media purchased from Invitrogen) and maintained under standard conditions at 37^o^C in a humidified atmosphere containing 5% CO_2_. Heterologous expression was performed by transfecting cells with 5 μg of plasmid cDNAs encoding for Ca_v_3.2 WT or R132H variant and empty pEGFP vector as transfection marker using the calcium/phosphate method.

***Patch clamp electrophysiology***

Patch clamp recordings of T-type currents in tsA-201 cells were performed 72 h after transfection in the whole-cell configuration at room temperature (22-24 °C) in a bath solution containing (in millimolar): 5 BaCl_2_, 5 KCl, 1 MgCl_2_, 128 NaCl, 10 TEA-Cl, 10 D-glucose, 10 4-(2-hydroxyethyl)-1-piperazineethanesulfonic acid (HEPES) (pH 7.2 with NaOH unless stated otherwise). Patch pipettes were filled with a solution containing (in millimolar): 110 CsCl, 3 Mg-ATP, 0.5 Na-GTP, 2.5 MgCl_2_, 5 D-glucose, 10 EGTA, and 10 HEPES (pH 7.4 with CsOH), and had a resistance of 2–4 MΩ. The linear leak component of the current was corrected using a P/4 subtraction protocol and current traces were digitized at 10 kHz and filtered at 2 kHz. The voltage dependence of activation was determined by measuring the peak of the T-type current in response to depolarizing steps from -90 mV to +30 mV applied every 5 sec from a holding membrane potential of -100 mV. The current-voltage relationship (*I*/*V*) curve was fitted with the following modified Boltzmann equation (1):

$$\left( 1 \right) I\left( V \right)= Gmax \frac{(V-Vrev)}{1+ \exp\frac{(V0.5-V)}{k}}$$

with *I*(*V*) being the peak current amplitude at the command potential *V*, *G*_rev_ the maximum conductance, *V_rev_* the reversal potential, *V*_0.5_ the half-activation potential, and *k* the slope factor. The voltage dependence of the whole-cell T-type channel conductance was calculated using the following modified Boltzmann equation (2):

$$\left( 2 \right) G\left( V \right)= \frac{Gmax}{1+ \exp\frac{(V0.5-V)}{k}}$$

with *G*(*V*) being the T-type channel conductance at the command potential *V*. The voltage dependence of the steady-state inactivation of Ca_v_3.2 channels was determined by measuring the peak T-type current amplitude in response to a 150 ms depolarizing step to -20 mV applied after a 5 s-long conditioning prepulse ranging from -120 mV to -30 mV. The current amplitude obtained during each test pulse was normalized to the maximal current amplitude and plotted as a function of the prepulse potential. The voltage dependence of the steady-state inactivation was fitted with the following two-state Boltzmann function (3):

$$\left( 3 \right) I\left( V \right)= \frac{Imax}{1+ \exp\frac{(V-V0.5)}{k}}$$

with *I*_max_ corresponding to the maximal peak current amplitude and *V*_0.5_ to the half-inactivation voltage. The recovery from inactivation was assessed using a double-pulse protocol from a holding potential of -100 mV. The cell membrane was depolarized for 2 s at 0 mV (inactivating prepulse) to ensure complete inactivation of the channel, and then to -20 mV for 150 ms (test pulse) after an increasing time period (interpulse) ranging between 0.1 ms and 7 s at -100 mV. The peak current from the test pulse was plotted as a ratio of the maximum prepulse current versus interpulse interval. The recovery from inactivation curve was fitted with the following single-exponential function (4):

$$\left( 4 \right) \frac{I}{Imax}=A \times(1- exp\frac{-t}{\tau})$$

where τ is the time constant for channel recovery from inactivation.

All recordings were performed using an Axopatch 200B amplifier (Axon Instruments) and acquisition and analysis were performed using pClamp 10 and Clampfit 10 softwares, respectively (Axon Instruments).

***Statistical analysis***

Data values are presented as mean ± S.E.M for n measurements. Statistical analysis was performed using a two-tailed Student’s *t* test with GraphPad Prism 7 and datasets were considered significantly different for *p*<0.05.
